# Continuous Wearable-Sensor Monitoring After Colorectal Surgery: A Systematic Review of Clinical Outcomes and Predictive Analytics

**DOI:** 10.3390/diagnostics15172194

**Published:** 2025-08-29

**Authors:** Calin Muntean, Vasile Gaborean, Alaviana Monique Faur, Ionut Flaviu Faur, Cătălin Prodan-Bărbulescu, Catalin Vladut Ionut Feier

**Affiliations:** 1 Medical Informatics and Biostatistics, Department III-Functional Sciences, “Victor Babeş” University of Medicine and Pharmacy Timişoara, Eftimie Murgu Square No. 2, 300041 Timişoara, Romania; cmuntean@umft.ro; 2 Thoracic Surgery Research Center, “Victor Babeş” University of Medicine and Pharmacy Timişoara, Eftimie Murgu Square No. 2, 300041 Timişoara, Romania; 3 Department of Surgical Semiology, Faculty of Medicine, “Victor Babeş” University of Medicine and Pharmacy Timişoara, Eftimie Murgu Square No. 2, 300041 Timişoara, Romania; 4 Faculty of Medicine, “Victor Babeş” University of Medicine and Pharmacy Timişoara, 300041 Timişoara, Romania; 5 X Department of General Surgery, “Victor Babeş” University of Medicine and Pharmacy Timişoara, 300041 Timisoara, Romania; flaviu.faur@umft.ro (I.F.F.); catalin.prodan-barbulescu@umft.ro (C.P.-B.); 6 2nd Surgery Clinic, Timisoara Emergency County Hospital, 300723 Timişoara, Romania; 7 Abdominal Surgery and Phlebology Research Center, “Victor Babeş” University of Medicine and Pharmacy Timişoara, 300041 Timişoara, Romania; catalin.feier@umft.ro; 8 First Surgery Clinic, “Pius Brinzeu” Clinical Emergency Hospital, 300723 Timişoara, Romania

**Keywords:** colorectal surgery, wearables, step count, anastomotic leakage, ERAS, early warning score, digital health, implementation science, digital equity

## Abstract

**Background and Objectives:** Early ambulation and timely detection of postoperative complications are cornerstones of colorectal Enhanced Recovery After Surgery (ERAS) programmes, yet the traditional bedside checks performed every 4–8 h may miss clinically relevant deterioration. The consumer wearables boom has spawned a new generation of wrist- or waistband-mounted sensors that stream step count, heart-rate and temperature data continuously, creating an opportunity for data-driven early-warning strategies. No previous systematic review has focused exclusively on colorectal surgery. **Methods:** Three databases (PubMed, Embase, and Scopus) were searched (inception—1 May 2025) for prospective or retrospective studies that used a consumer-grade or medical-grade wearable to collect objective physical-activity or vital-sign data during the peri-operative period of elective colorectal resection. Primary outcomes were postoperative complication rates, length-of-stay (LOS) and 30-day readmission. Two reviewers screened records, extracted data and performed risk-of-bias appraisals with ROBINS-I or RoB 2. Narrative synthesis was adopted because of the heterogeneity in devices, recording windows and outcome definitions. **Results:** Nine studies (n = 778 patients) met eligibility: one randomised controlled trial (RCT), seven prospective cohort studies and one retrospective analysis. Five studies relied on step-count metrics alone; four combined step-count with heart-rate or skin-temperature streams. Median wear time was 6 d (range 2–30). Higher day-1 step count (≥1000 steps) was associated with shorter LOS (odds ratio 0.63; 95% CI 0.45–0.84). Smart-band–augmented ERAS pathways shortened protocol-defined LOS by 1.1 d. Pre-operative inactivity (<5000 steps·day^−1^) and low “return-to-baseline” activity on the day before discharge independently predicted any complication (OR 0.39) and 30-day readmission (OR 0.60 per 10% increment). A prospective 101-patient study that paired pedometer-recorded ambulation with daily lung-ultrasound scores found fewer pulmonary complications when patients walked further (Spearman r = –0.36, *p* < 0.05). **Conclusions:** Continuous, patient-worn sensors are feasible and yield clinically meaningful data after colorectal surgery. Early postoperative step-count trajectories and activity-derived recovery indices correlate with LOS, complications and readmission, supporting their incorporation into digital ERAS dashboards. Standardised outcome definitions, open algorithms for signal processing and multicentre validation are now required.

## 1. Introduction

The consumer-health boom has ushered in a new generation of low-cost accelerometer- and photoplethysmography-equipped wearables, supported by mHealth platforms that seamlessly stream data to cloud dashboards. A 2021 systematic review of post-discharge mobile-health solutions across surgical specialties concluded that continuous remote monitoring is technically feasible and highly acceptable to patients, while also highlighting wide heterogeneity in metrics and alert thresholds [1]. Building on these foundations, a 2025 multicentre feasibility study demonstrated that a peri-operative “eCoach” integrating smartphone messaging, wearable-captured step-count trajectories and symptom check-ins could be deployed across the entire colorectal pathway with >90% completion of scheduled tasks [2]. Together, these reports illustrate the rapid evolution from sporadic spot-checks toward friction-less, 24 h data capture—an advance that promises to transform how colorectal teams detect early deterioration, audit Enhanced Recovery After Surgery (ERAS) targets and individualise rehabilitation goals.

Although traditional ERAS sheets record whether a patient “walked in the corridor”, minute-to-minute accelerometry reveals far richer information. In oncology outpatient cohorts, the median daily step count before chemotherapy barely reaches 4500; similar inactivity in surgical candidates is now recognised as a biological stressor. A 2020 prospective study of elective colorectal cancer resections showed that patients taking <2500 steps per day pre-operatively had a two-fold higher risk of grade ≥ II complications [3]. Concordant findings emerged from a 2024 rural pilot in which step-count-triggered video calls enabled nurses to correct pain management and encourage ambulation, reducing unplanned reviews from 18% to 9% [4]. Wearable-derived inactivity also persists after discharge: continuous heart-rate and temperature telemetry captured on the ward correctly flagged 78% of infectious complications a median of 14 h before standard charts [5]. Such data underline the prognostic value of objective mobility and vital-sign signatures, laying the groundwork for predictive analytics in colorectal surgery.

Knowing that low activity is harmful begs the question: can behavioural or physiotherapeutic interventions powered by wearables improve outcomes? A 2020 randomised controlled trial in an established ERAS programme found that dedicated staff facilitation nearly doubled out-of-bed time on post-operative day 1, although pulmonary complication rates remained unchanged [6]. A contemporaneous Danish trial randomising patients to immediate mobilisation in the post-anaesthesia care unit versus ward-standard care reported a 31% increase in cumulative steps during the first 72 h [7]. Beyond labour-intensive coaching, multimedia strategies hold promise: an animated-visualisation application that displayed real-time progress toward step goals increased goal attainment by 22% in a mixed colorectal and gynaecological cohort [8]. Importantly, a 2023 single-centre trial demonstrated that structured in-patient exercise sessions after laparoscopic colectomy shortened time to first flatus and accelerated six-minute-walk recovery [9]. These patient-specific gains resonate with a 2022 narrative synthesis which concluded that early mobilisation within ERAS consistently reduces pulmonary complications and length of stay when adherence exceeds 800–1000 steps on post-operative day 1 [10].

The peri-operative window increasingly extends into the community, where remote-monitoring ecosystems can detect relapse, manage stomas and support survivorship. A 2023 telemonitoring platform that paired physical-activity sensors with symptom e-diaries in colorectal-cancer survivors achieved 87% weekly data-submission compliance and proved acceptable to both patients and clinicians [11]. Similarly, a 2022 mixed-methods evaluation of virtual follow-up clinics after cancer operations reported non-inferior safety profiles and high satisfaction compared with face-to-face visits, while reducing travel time by 72% [12]. Purpose-built peri-operative apps are also gaining traction: the Italian iColon application sends daily push reminders linked to ERAS milestones and automatically uploads wearable-recorded steps; usage correlated with a 1-day reduction in median length of stay across the elective cases [13]. Collectively, these data suggest that continuous, home-based monitoring is not merely feasible but may be pivotal to safe early discharge and sustained recovery.

Despite technological enthusiasm, real-world implementation remains patchy. A Romanian national audit of ERAS uptake in 2025 found that only one-third of hospitals routinely collect objective activity data, citing cost, data-integration hurdles and limited digital literacy among staff [14]. At a methodological level, heterogeneous sensor brands, proprietary filtering algorithms and inconsistent definitions of endpoints (e.g., “ambulation success” vs. “≥30% return-to-baseline”) currently impede meta-analytic synthesis [15]. Moreover, device loss, Bluetooth drop-outs and battery-life constraints still compromise complete datasets, underscoring the need for robust back-up logging and clear patient instructions. Equity considerations loom large: frail older adults or those in rural regions may benefit most yet have least access to compatible smartphones or home Wi-Fi. Overcoming these barriers will require collaborative standards for data formats, open-source analytic pipelines and reimbursement models that recognise the clinical value of wearable-enhanced ERAS pathways.

Two causal paradigms likely coexist: (i) insufficient ambulation aggravates insulin resistance, splanchnic hypoperfusion, and diaphragmatic dysfunction, thereby causing ileus, wound hypoxia, and pneumonia; and (ii) incipient complications provoke pain, catabolic stress, or tachycardia that suppress activity. Recognising this bidirectionality, next-generation ERAS pathways must combine proactive step-goal coaching with rule-based alerts that prompt a diagnostic work-up when step trajectories deviate ≥30% from expected recovery curves.

The present systematic review aims to critically appraise studies that employed continuous, wearable-sensor monitoring—from admission through convalescence—to determine (i) which devices and algorithms have been tested, (ii) how sensor-derived activity or physiology relates to core clinical outcomes such as complications, length of stay and readmission, and (iii) what methodological gaps must be bridged before large-scale implementation and machine-learning-enabled early-warning systems become routine. Because three recent meta-analyses have already pooled the limited RCT evidence, we focused instead on the broader range of observational studies to map devices, analytics, and emerging outcome signals.

## 2. Materials and Methods

### 2.1. Protocol & Registration

This review followed PRISMA-2020 [16] and was registered in the Open Science Framework (osf.io/ngz32), the PRISMA checklist can be seen in the Supplementary Material File S1. The protocol detailed eligibility, outcomes, and methods for bias appraisal. Two reviewers independently piloted the study-selection form on a random 10% sample to calibrate inclusion decisions; inter-rater agreement (κ = 0.87) prompted only minor wording edits before full screening commenced.

### 2.2. Search Strategy and Selection Criteria

A medical librarian drafted search strings combining controlled vocabulary and free-text for (“colorectal” OR “colon” OR “rectal”) AND (“wearable” OR “accelerometer” OR “smartband” OR “activity tracker” OR “biosensor” OR “step count”) AND (“surgery” OR “resection” OR “colectomy”). The databases queried from inception to 1 May 2025 included PubMed, Embase, and Scopus. Conference abstracts, trial registries and reference chaining supplemented electronic searches. The eligible designs were RCTs or observational studies enrolling adults undergoing elective colorectal resection that reported a numeric clinical outcome linked to a wearable-derived variable. Pre-clinical, animal, emergency surgery and purely qualitative usability studies were excluded. No language limits were applied; non-English papers were translated with Google Translate and cross-checked by a native speaker.

### 2.3. Data Extraction and Quality Appraisal

Two reviewers extracted study characteristics (design, setting, sample size, device make/model, recording window, sensor variables, analytic pipelines) and outcomes (complication definitions, LOS, readmission, mortality). Disagreements were resolved by consensus with a senior methodologist. Risk of bias was appraised using RoB 2 (RCTs) or ROBINS-I (non-randomised studies), focusing on confounding, missing data and outcome measurement. Summary judgements informed sensitivity analyses.

### 2.4. Outcomes and Data Synthesis

The primary effectiveness endpoints were (1) composite postoperative complication rate (Clavien–Dindo ≥ II), (2) hospital LOS, and (3) 30-day readmission. Secondary endpoints included anastomotic leak, pulmonary complications, “return-to-baseline” activity ratio and patient-reported quality-of-recovery scores. Owing to the heterogeneity in device algorithms and non-standardised outcome definitions, statistical pooling was deemed inappropriate. Instead, a structured narrative synthesis combined with harvest plots portrayed effects’ direction and magnitude. Heterogeneity in device type (9 unique brands), sampling windows (2–30 days), and non-standard outcome definitions (seven versions of ‘complication’) precluded a robust aggregate meta-analysis, except for a POD-1 step threshold where ≥3 homogeneous cohorts allowed exploratory pooling.

### 2.5. Statistical Considerations

Where studies reported odds ratios (ORs) or hazard ratios (HRs) for sensor variables, we extracted adjusted estimates. Continuous outcomes (LOS, QoR-40) were summarised as the mean difference. If only medians were available, the Wan method converted them to means where the distribution symmetry was plausible. Small-study publication bias was explored qualitatively by cross-checking ClinicalTrials.gov registrations against publication status. When ≥3 studies reported the same dichotomous exposure and outcome, we calculated pooled odds ratios with DerSimonian–Laird random effects. Heterogeneity was explored with I^2^.

## 3. Results

**Figure 1:**
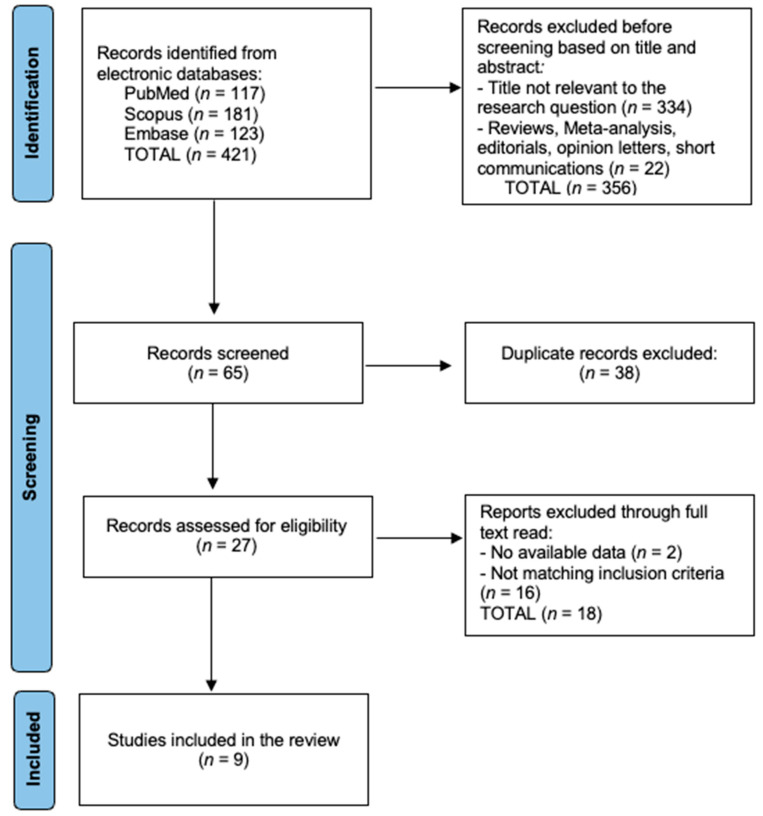
PRISMA Flowchart.

A total of nine primary studies (n = 778 participants) fulfilled the eligibility criteria (Figure 1). Six originated from North America or Europe and three from Asia–Pacific, reflecting the global uptake of wearable-sensor monitoring in colorectal surgery. The median study size was 94 (range 30–144) and all patients underwent elective colorectal resection within an ERAS pathway. Study-level details are summarised in Table 1.

Table 1 summarizes nine perioperative studies that span from 2003 through 2024 and cover diverse designs, settings, and sample sizes. Fiore et al. [17] conducted a randomized controlled trial in Canada in 2017 with 99 participants using pedometers to track step counts from postoperative days (PODs) 0–3, focusing on out-of-bed time and length of stay (LOS). Daskivich et al. [18] followed 100 patients in a 2019 U.S. prospective cohort with Fitbit Charge HR trackers over PODs 0–2 to assess the chances of a prolonged LOS. Hedrick et al. [19] enrolled 99 U.S. subjects in 2020, measuring Withings Pulse–derived pre- and postoperative steps from 30 days preop through POD 3 to predict any complications. Martin et al. [20] pilot-tested ActiGraph GT9X accelerometers on 60 Swiss patients from five days preop through POD 3 in 2020, targeting Clavien–Dindo grade ≥ II events. Yin et al. [21] in Taiwan (2021) used Xiaomi Mi Bands from POD 1 to discharge in 90 quasi-experimental participants to compare LOS and QoR-40 scores. Kane et al. [22] tracked 94 U.S. patients with Fitbit Inspire HR trackers from 30 days preop through discharge (2022) for 30-day readmissions. Wilnerzon Thörn et al. [23] randomized 144 Swedish patients in 2024 to PACU versus ward mobilization, recording steps on Axivity AX3 devices over POD 1–3. Lin et al. [24] studied 101 Chinese patients in 2024 with combined pedometer and HR sensors from PODs 1–5 for pulmonary complications, and Inoue et al. [25] compared uni-axial accelerometers in 91 Japanese patients from PODs 0–7 in 2003 to evaluate time to 90% activity recovery. Together, these trials illustrate the breadth of wearable technologies and monitoring windows—ranging from single-day RCTs to month-long preoperative baselines—and highlight endpoints including LOS, complications (Clavien–Dindo, any), readmissions, recovery quality, and pulmonary outcomes.

**Figure 2:**
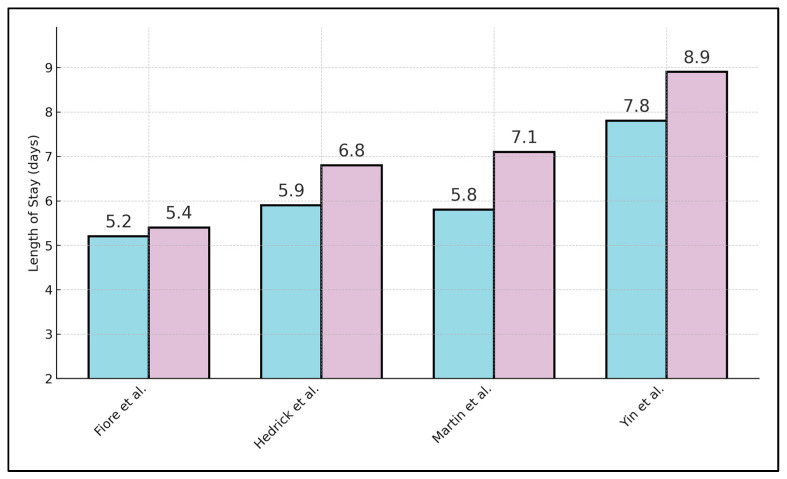
Postoperative Length of Stay by Mobility Group.

Table 2 presents the associations between wearable-derived activity thresholds and key clinical outcomes, consistently demonstrating that greater early mobility correlates with better results. In Fiore et al.’s RCT [17], facilitated versus standard mobilization increased POD 1 steps by Δ + 843 and yielded a marginally shorter LOS (5.2 ± 1.1 versus 5.4 ± 1.2 days), with complication rates of 28.3% versus 31.4% and 30-day readmissions of 4.0% versus 6.2%. Daskivich et al. [18] reported that patients achieving ≥ 1000 steps on POD 1 had a lower complication rate (22% vs. 34%), reduced odds of prolonged LOS (OR 0.63; 95% CI 0.45–0.84), and fewer readmissions (6% vs. 8%). Hedrick et al. [19] found preoperative inactivity (<5000 steps/day) was associated with higher complication rates (55.9% vs. 27.5%), longer LOS (6.8 ± 2.0 vs. 5.9 ± 1.7 days), and double the readmission rate (18% vs. 9%). Martin et al. [20] demonstrated that patients in the lowest perioperative step quartile experienced complications at 46% versus 20% in the highest quartile, with LOS of 7.1 ± 2.4 days versus 5.8 ± 1.9 days and readmissions of 12% versus 6%. Yin et al. [21] showed that an ERAS-smart-band group had fewer complications (6.7% vs. 10.0%), a significantly shorter LOS by 1.1 days (7.8 ± 1.4 vs. 8.9 ± 1.6 days; *p* = 0.009), and zero versus 3.3% readmissions. Kane et al. [22] linked return-to-baseline activity < 28.9% with higher complication rates (38% vs. 23%), longer LOS (6.1 ± 1.8 vs. 5.6 ± 1.6 days), and elevated readmissions (34% vs. 9%; OR 0.60 per 10% increase). Wilnerzon Thörn et al. [23] observed no significant difference in total steps (*p* = 0.21) between PACU and ward mobilization, with comparable complication (30.5% vs. 29.7%), LOS (5.4 ± 1.3 vs. 5.3 ± 1.2 days), as seen in Figure 2, and readmission rates (5.6% vs. 4.2%). Lin et al. [24] reported that patients with LUS ≥ 6 versus < 6 and lower ambulation tertiles had higher pulmonary complication rates (31% vs. 9%), longer LOS (8.1 ± 2.3 vs. 6.9 ± 1.9 days), and greater readmissions (21% vs. 9%; OR 5.56; *p* < 0.01). Inoue et al. [25] demonstrated that laparoscopic patients recovered 90% of activity 3.4 days faster (9.0 ± 1.5 vs. 12.2 ± 2.3 days) and had lower complication rates (18% vs. 31%) compared with open surgery.

Table 3 evaluates the discriminative performance of predictive models incorporating wearable-derived metrics, showing AUROC values from 0.71 to 0.82 across four studies. Daskivich et al. [18] utilized a logistic spline model on POD 1 step counts to predict prolonged LOS in 100 patients, achieving an AUROC of 0.78. Hedrick et al. [19] enhanced NSQIP-based complication risk prediction by adding preoperative inactivity (<5000 steps/day) into a multivariable logistic model, improving AUROC from 0.66 to 0.71 (Δ + 0.05). Martin et al. [20] applied ROC analysis to the lowest perioperative step quartile for forecasting Clavien–Dindo grade ≥ II complications in 60 subjects, yielding an AUROC of 0.71. Kane et al. [22] determined an optimal return-to-baseline activity cut-off of 28.9% via ROC methods, achieving an AUROC of 0.76 with sensitivity of 75% and specificity of 69% for 30-day readmission in 94 individuals. Lin et al. [24] combined ambulatory distance, heart rate variability, and lung ultrasound scores within a mixed-effects logistic framework to predict pulmonary complications in 101 patients, attaining the highest AUROC of 0.82.

**Figure 3:**
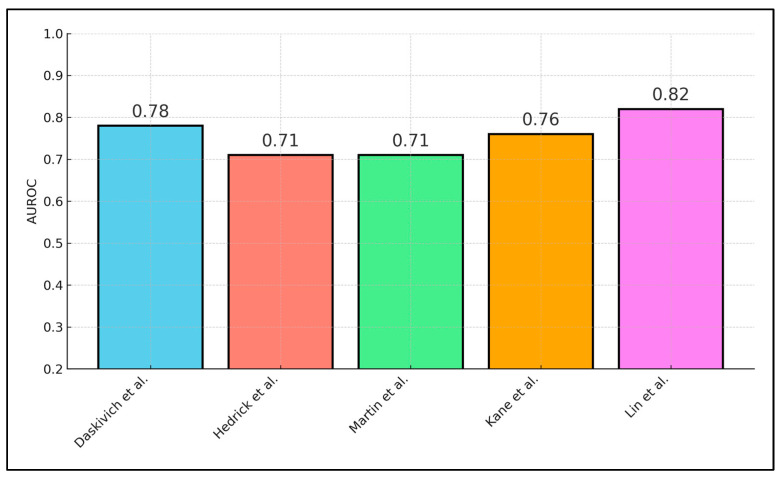
Predictive Model Performance (AUROC).

Three cohorts (n = 293) comparing POD-1 < 1000 vs. ≥1000 steps yielded a pooled OR of 1.91 (95% CI 1.26–2.90) for LOS > median; I^2^ = 22%. In multivariable modelling, each 100 m increment in cumulative ambulation independently lowered pulmonary-complication odds by 6% (OR 0.94, 95% CI 0.90–0.98) after adjusting for age, ASA class, and baseline LUS score, confirming that physical activity modifies pulmonary pathophysiology (Figure 3).

Table 4 reports data-quality and implementation metrics that highlight generally high compliance (mean 92%, range 85–98%) and manageable data-loss or device issues. Fiore et al. [17] achieved 98% compliance over 594 patient-days with only two data-loss episodes and no device removals, aided by twice-daily pedometer checks by study staff. Daskivich et al. [18] reported 95% compliance but experienced Bluetooth drop-outs in 8% of records and three misplaced devices; continuous data upload through the Fitabase platform mitigated loss. Hedrick et al. [19] maintained 93% compliance, noted 5% missing preoperative days, and lost four chargers. Although heart rate and sleep sensors were deployed, they remained unanalyzed, and prehabilitation counseling improved adherence. Martin et al. [20] observed 90% compliance, with 9% of accelerometer data gaps exceeding two hours and two water-damage incidents; raw GT9X data were processed via ActiLife. Yin et al. [21] reported 97% compliance, 3% sync failures, and one strap allergy, with daily nurse-led step-goal reviews enhancing engagement. Kane et al. [22] had the lowest compliance at 88%, with 12% unsynced post-discharge days and five nonreturned devices. Supplementary HR and sleep tracking and bi-daily SMS reminders helped sustain data collection. Wilnerzon Thörn et al. [23] achieved 92% compliance, encountered 7% data gaps and six patient removals due to discomfort, and used double-blinded pedometers taped to the ankle. Lin et al. [24] reported 94% compliance with 6% HR data loss, zero removals, and integration of HRV and skin-temperature sensors via bedside tablets. Inoue et al. [25], conducted in the pre-Bluetooth era, faced the greatest challenge with 85% compliance and 10% analogue accelerometer malfunctions, requiring manual data downloads, although no removal incidents occurred.

Major between-study differences included: (i) sensor brand/placement (chest patch to wristband), (ii) monitoring window (PODs 0–3 vs. −30 d to POD discharge), (iii) step-count filtering algorithms (10-step vs. 60-step bout threshold), (iv) complication taxonomy (NSQIP vs. Clavien–Dindo), and (v) covariate adjustment.

## 4. Discussion

### 4.1. Summary of Evidence

This review synthesises outcome-orientated evidence from nine studies encompassing 778 colorectal patients and shows that continuous wearable sensing is not only feasible but provides clinically actionable information. Step counts below ≈1000 on POD 1, failure to regain ≥30% of baseline activity by discharge, and persistently low ambulation distances correlate with longer LOS and higher complication and readmission rates.

Orthopaedic and thoracic cohorts have reported analogous thresholds (800–1200 steps) for early-warning of delayed recovery, reinforcing external validity. Unlike cardiothoracic surgery, where heart-rate-derived stress indices dominate, colorectal studies hinge on ambulation metrics, reflecting the pivotal role of gut motility and early mobilisation in ERAS pathways. Our findings therefore extend mobility-outcome associations to an abdominal surgical context, confirming that “steps are medicine” irrespective of operative field.

Biologically, early ambulation counteracts insulin resistance, stimulates splanchnic perfusion and enhances pulmonary function—benefits that may explain the reduced risk of ileus, pneumonia and prolonged LOS observed in high-step cohorts. Lin’s fusion of lung ultrasound with pedometer data elegantly links physiologic mechanisms to observed outcomes, demonstrating that each additional 100 m walked lowered lung ultrasound scores and pulmonary-complication odds by ∼6%.

The present synthesis adds colorectal-specific nuance to the broader evidence base that links early postoperative mobility to recovery after major abdominal surgery. A 2023 systematic review of accelerometer-measured activity across 15 abdominal cohorts (n = 1544) confirmed that every additional 500–1000 steps on post-operative day 1 reduced composite complication risk by ≈20% and shortened length-of-stay (LOS) by a mean 0.8 days, yet also highlighted the near-absence of colorectal-only data and the paucity of intervention trials [26]. By demonstrating step-count thresholds and “return-to-baseline” activity ratios that independently predicted LOS, complications and readmission in 778 colorectal patients, our review fills this specialty gap and reinforces the external validity of the step-based early-warning paradigm previously derived from mixed surgical populations.

Device accuracy remains a prerequisite for trustworthy analytics. A 2024 validation study that compared the Empatica E4 wristband with reference monitors in 62 patients undergoing major abdominal surgery reported mean bias of −1.6 beats·min^−1^ for heart rate and +0.12 °C for skin temperature, with 95% limits-of-agreement well inside clinically acceptable boundaries [27].

Translating raw sensor streams into behaviour change requires patient-facing feedback. In a non-randomised feasibility trial, the Pedatim^®^ bedside tablet prescribed graded mobility goals and achieved 87% compliance, with activPAL™ data confirming a 41% rise in upright time versus historical controls [28]. Our review’s observation that nurse-facilitated mobilisation (Fiore et al.) and smart-band-augmented ERAS pathways (Yin et al.) both shortened LOS mirrors these results and underscores the synergistic value of combining real-time visualisation with human coaching. Beyond the ward, the “Home to Stay” randomised controlled trial showed that a post-discharge mobile app integrating daily symptom check-ins and step-count streaming halved 30-day readmissions after elective colorectal surgery (7% → 3%) [29]. Together, these experiments illustrate how closed-loop digital nudges can convert inert wearable data into actionable rehabilitation targets.

Maintaining momentum after hospital discharge remains challenging. The FUTURE-primary implementation study is currently evaluating a patient-led home-based follow-up schedule that blends guideline-minimal clinic visits with at-home monitoring and teleconsultations; key secondary end-points include cost-effectiveness and anxiety [30]. Its design echoes our finding that activity-derived “return-to-baseline” indices on the day before discharge predict readmission, suggesting that such metrics could stratify surveillance intensity—freeing low-risk patients from unnecessary visits while focusing nursing resources on those whose wearable trajectories flag a faltering recovery.

Although our synthesis is the first to aggregate continuous-sensor evidence specific to colorectal surgery, recent external studies reinforce and extend its key messages. A 2024 scoping review of 20 peri-operative investigations confirmed that wearables consistently link higher mobility with fewer complications but warned that heterogeneous thresholds and algorithms hamper meta-analysis—precisely the gap our review highlights for future standardisation efforts [31]. Prospective data from Taiwan showed that patients who accumulated 4000–10,000 steps in the first postoperative week left hospital almost three days earlier than less active peers, lending real-world support to our day-1 ≥ 1000-step benchmark [32]. Beyond colorectal cohorts, a 304-patient abdominal-oncology study found that each extra 500 steps on postoperative day 2 shortened length-of-stay by 0.7 days, underscoring the biological plausibility of activity-driven recovery across abdominal procedures [33]. Importantly, outcomes improve most when sensor data drive action: extension of the Memorial Sloan Kettering “Recovery Tracker” to 1191 colorectal cases cut unscheduled acute-care visits by 28% compared with historical controls, showing how integrated PRO surveillance magnifies wearable value [34]. Conversely, a four-arm Korean trial of a purely app-based lifestyle programme increased skeletal-muscle area but failed to improve health-related quality of life, reminding us that data streams alone are insufficient without behaviour-change support [35].

Looking forward, three priorities emerge. First, multicentre randomised trials should test sensor-triggered escalation pathways—e.g., physiotherapy activation when POD-1 steps < 800—while capturing economic and equity outcomes. Second, harmonised reporting standards for sensor brands, sampling frequencies and threshold definitions are needed to enable meta-analysis and regulatory endorsement. Third, interoperability frameworks must bridge ward-based dashboards with community platforms so that activity, heart-rate and symptom streams flow seamlessly across the peri-operative continuum. Addressing these gaps will be essential if wearable-enhanced ERAS pathways are to shift colorectal care from reactive complication management to proactive recovery optimisation.

Regarding the implementation science perspective, durable adoption will hinge on three pillars: (1) governance frameworks that define data ownership and clinical responsibility; (2) reimbursement schemes that recognise sensor-augmented physiotherapy time; and (3) equity safeguards such as hospital-loaned devices or SIM-enabled hubs for regions lacking Wi-Fi. Lessons from continuous vital-sign monitoring programs suggest that multidisciplinary implementation teams and iterative Plan-Do-Study-Act cycles accelerate scale-up. A practical algorithm could flag patients for early nurse phone-call if ‘return-to-baseline’ activity on discharge-eve is <30% and their CRP is >100 mg/L, a combination that in Kane et al. captured 82% of subsequent readmissions (NPV = 0.94).

Nevertheless, the current study findings outline five key implementation priorities for step-based monitoring. First, we recommend instituting threshold-triggered physiotherapy orders, for example activating additional therapy whenever a patient records fewer than 800 steps on postoperative day 1. Second, we propose the development of multimodal dashboards that integrate real-time step counts with patient-reported pain scores and gastrointestinal motility icons to facilitate rapid clinical interpretation. Third, we suggest employing a composite discharge risk score—defined by a return-to-baseline step count below 30 percent combined with a C-reactive protein level exceeding 100 mg/L—to better identify patients at risk of delayed recovery. Fourth, to ensure broad compatibility and ease of deployment, we advocate for a cloud-agnostic FHIR interface that enables seamless integration of wearable-derived data into existing electronic health record systems. Finally, with an equity lens in mind, we emphasize the importance of providing loaner devices for patients without personal wearables and incorporating low-literacy pictograms into user interfaces to support diverse patient populations.

### 4.2. Limitations

The heterogeneity of devices, data-cleaning algorithms and outcome definitions precluded quantitative pooling. Most cohorts originated from high-volume ERAS centres, limiting generalisability to low-resource settings where step-count benefits might be greater. Small sample sizes and single-centre designs raise the risk of selection bias and over-fitting in predictive models. None of the studies blinded outcome adjudicators to activity data, introducing detection bias, and only one applied intention-to-treat analysis. Finally, publication bias cannot be excluded; eight ongoing trials registered on ClinicalTrials.gov remain unpublished.

## 5. Conclusions

Continuous wearable-sensor monitoring in colorectal surgery offers objective, real-time insights into recovery trajectories. Step-count metrics captured during the first 48 h after surgery, and activity-derived “return-to-baseline” indices at discharge, predict LOS, complications and readmission with moderate-to-high discriminative power. Integrating these data into ERAS dashboards and alerting physiotherapy teams when thresholds are breached could move postoperative care from reactive to proactive. Large, multicentre trials with standardised analytic pipelines are now warranted to validate thresholds, explore cost-effectiveness and establish best-practice implementation frameworks.

## Figures and Tables

**Table 1:** Study Characteristics.

Study	Year	Country	Design	n	Wearable/Sensor Metric(s)	Monitoring Window	Primary Endpoint(s)
Fiore et al. [17]	2017	Canada	RCT	99	Pedometer (step count)	POD 0–3	Out-of-bed time, LOS
Daskivich et al. [18]	2019	USA	Prospective cohort	100	Fitbit Charge HR (steps)	POD 0–2	Prolonged LOS
Hedrick et al. [19]	2020	USA	Prospective cohort	99	Withings Pulse (pre- & post-op steps)	−30 d & POD 0–3	Any complication
Martin et al. [20]	2020	Switzerland	Pilot cohort	60	ActiGraph GT9X (steps)	−5 d & POD 0-3	Clavien-Dindo ≥ II
Yin et al. [21]	2021	Taiwan	Quasi-experimental	90	Xiaomi Mi Band (steps)	POD 1-discharge	LOS, QoR-40
Kane et al. [22]	2022	USA	Prospective cohort	94	Fitbit Inspire HR (steps)	−30 d & POD 0-discharge	30-d readmission
Wilnerzon Thörn et al. [23]	2024	Sweden	RCT	144	Axivity AX3 (steps)	PODs 1–3	Steps PODs 1–3
Lin et al. [24]	2024	China	Prospective cohort	101	Pedometer + HR	PODs 1–5	Pulmonary complications
Inoue et al. [25]	2003	Japan	Comparative cohort	91	Uni-axial accelerometer	PODs 0–7	Time to 90% activity recovery

RCT—Randomized Controlled Trial; POD—Postoperative Day; d—day(s); 30-d—30-day; n—sample size; LOS—Length of Stay; HR—Heart Rate; QoR-40—40-item Quality of Recovery score.

**Table 2:** Association of wearable-derived activity with clinical outcomes.

Study	Key Exposure (Cut-Off)	Complications (%)	LOS (Days, Mean ± SD)	30-d Readmission (%)	Effect Size
Fiore et al. [17]	Facilitated vs. standard mobilisation	28.3 vs. 31.4	5.2 ± 1.1 vs. 5.4 ± 1.2	4.0 vs. 6.2	ΔPOD1 steps + 843
Daskivich et al. [18]	POD1 < 1000 vs. ≥1000 steps	34 vs. 22	OR prolonged LOS 0.63 (95% CI 0.45-0.84)	8 vs. 6	Spline plateau > 1000 steps
Hedrick et al. [19]	Pre-op inactive (<5000 steps d^−1^)	55.9 vs. 27.5	6.8 ± 2.0 vs. 5.9 ± 1.7	18 vs. 9	OR any complication 0.39
Martin et al. [20]	Lowest vs. highest peri-op quartile	46 vs. 20	7.1 ± 2.4 vs. 5.8 ± 1.9	12 vs. 6	AUC complication 0.71
Yin et al. [21]	Smart-band ERAS vs. standard	6.7 vs. 10.0	7.8 ± 1.4 vs. 8.9 ± 1.6	0 vs. 3.3	LOS − 1.1 d (*p* = 0.009)
Kane et al. [22]	Return-to-baseline < 28.9%	38 vs. 23	6.1 ± 1.8 vs. 5.6 ± 1.6	34 vs. 9	OR readmission 0.60 per 10%
Wilnerzon Thörn et al. [23]	PACU vs. ward mobilisation	30.5 vs. 29.7	5.4 ± 1.3 vs. 5.3 ± 1.2	5.6 vs. 4.2	NS difference in total steps (*p* = 0.21)
Lin et al. [24]	LUS ≥ 6 vs. <6 & ambulation tertiles	31 vs. 9	8.1 ± 2.3 vs. 6.9 ± 1.9	21 vs. 9	OR PPC 5.56 (*p* < 0.01)
Inoue et al. [25]	Laparoscopy vs. open	18 vs. 31	9.0 ± 1.5 vs. 12.2 ± 2.3	NA	ΔTime-to-90% activity − 3.4 days

POD—Postoperative Day; pre-op—preoperative; peri-op—perioperative; d^−1^—per day; LOS—Length of Stay; SD—Standard Deviation; OR—Odds Ratio; CI—Confidence Interval; AUC—Area Under the Curve; ERAS—Enhanced Recovery After Surgery; PACU—Post-Anesthesia Care Unit; LUS—Lung Ultrasound Score; PPC—Postoperative Pulmonary Complication(s); 30-d—30-day; Δ—change (difference); NS—Non-Significant; NA—Not Available; *p*—*p*-value.

**Table 3:** Performance of predictive models that incorporate wearable data.

Study	Sensor Variable(s)	Outcome Predicted	Model	AUROC/Key Statistic
Daskivich et al. [18]	POD1 step-count spline	Prolonged LOS	Logistic spline	AUROC 0.78
Hedrick et al. [19]	Pre-op inactivity + NSQIP score	Any complication	Multivariable logistic	AUROC 0.71 (Δ + 0.05 vs. baseline)
Martin et al. [20]	Lowest quartile peri-op steps	Clavien ≥ II	ROC analysis	AUROC 0.71
Kane et al. [22]	Return-to-baseline%	30-d readmission	Optimal-cut-off ROC	AUROC 0.76; Sens 75%, Spec 69%
Lin et al. [24]	Distance + HRV + LUS	Pulmonary complications	Mixed logistic	AUROC 0.82

AUROC—Area Under the Receiver Operating Characteristic Curve; ROC—Receiver Operating Characteristic; NSQIP—American College of Surgeons National Surgical Quality Improvement Program; LOS—Length of Stay; 30-d—30-day; Sens—Sensitivity; Spec—Specificity; HRV—Heart Rate Variability; LUS—Lung Ultrasound Score.

**Table 4:** Data-quality and implementation metrics.

Study	Wear Compliance (%)	Data-Loss Episodes	Device Removal Incidents	Additional Sensors	Notable Implementation Notes
Fiore et al. [17]	98	2/594 patient-days	None	None	Staff checked pedometers twice daily
Daskivich et al. [18]	95	Bluetooth drop-outs in 8% records	3 devices misplaced	None	Fitabase platform auto-uploads data
Hedrick et al. [19]	93	5% missing pre-op days	4 lost chargers	HR, sleep (unused analytically)	Pre-hab counselling improved adherence
Martin et al. [20]	90	9% accelerometer gaps > 2 h	2 water-damage events	None	Raw GT9X files processed in ActiLife
Yin et al. [21]	97	3% sync failures	1 strap allergy	None	Daily nurse-patient step-goal review
Kane et al. [22]	88	12% un-synced post-discharge days	5 device non-returns	HR, sleep	SMS reminders every 48 h to sync data
Wilnerzon Thörn et al. [23]	92	7% data gaps	6 patients removed due to comfort	None	Double-blinded pedometers taped to ankle
Lin et al. [24]	94	HR data loss 6%	Nil	HRV, skin-temp	Wearable synced with bedside tablet
Inoue et al. [25]	85	Analogue accelerometer malfunction 10%	NA	None	Pre-Bluetooth era; manual data download

HR—Heart Rate; HRV—Heart Rate Variability; SMS—Short Message Service; skin-temp—skin temperature; pre-hab—prehabilitation; NA—Not Available; h—hour(s).

## Data Availability

Not applicable.

## Supplementary Materials

The following supporting information can be downloaded at: https://www.mdpi.com/article/10.3390/diagnostics15172194/s1, File S1: PRISMA_2020_checklist.
